# Glial Cell Line-Derived Neurotrophic Factor (*GDNF*) as a Novel Candidate Gene of Anxiety

**DOI:** 10.1371/journal.pone.0080613

**Published:** 2013-12-06

**Authors:** Eszter Kotyuk, Gergely Keszler, Nora Nemeth, Zsolt Ronai, Maria Sasvari-Szekely, Anna Szekely

**Affiliations:** 1 Doctoral School of Psychology, Eötvös Loránd University, Budapest, Hungary; 2 Institute of Psychology, Eötvös Loránd University, Budapest, Hungary; 3 Department of Medical Chemistry, Molecular Biology and Pathobiochemistry, Semmelweis University, Budapest, Hungary; NIDCR/NIH, United States of America

## Abstract

Glial cell line-derived neurotrophic factor (GDNF) is a neurotrophic factor for dopaminergic neurons with promising therapeutic potential in Parkinson's disease. A few association analyses between GDNF gene polymorphisms and psychiatric disorders such as schizophrenia, attention deficit hyperactivity disorder and drug abuse have also been published but little is known about any effects of these polymorphisms on mood characteristics such as anxiety and depression. Here we present an association study between eight (rs1981844, rs3812047, rs3096140, rs2973041, rs2910702, rs1549250, rs2973050 and rs11111) GDNF single nucleotide polymorphisms (SNPs) and anxiety and depression scores measured by the Hospital Anxiety and Depression Scale (HADS) on 708 Caucasian young adults with no psychiatric history. Results of the allele-wise single marker association analyses provided significant effects of two single nucleotide polymorphisms on anxiety scores following the Bonferroni correction for multiple testing (p = 0.00070 and p = 0.00138 for rs3812047 and rs3096140, respectively), while no such result was obtained on depression scores. Haplotype analysis confirmed the role of these SNPs; mean anxiety scores raised according to the number of risk alleles present in the haplotypes (p = 0.00029). A significant sex-gene interaction was also observed since the effect of the rs3812047 A allele as a risk factor of anxiety was more pronounced in males. In conclusion, this is the first demonstration of a significant association between the GDNF gene and mood characteristics demonstrated by the association of two SNPs of the GDNF gene (rs3812047 and rs3096140) and individual variability of anxiety using self-report data from a non-clinical sample.

## Introduction

Glial cell line-derived neurotrophic factor (GDNF), a member of the TGFβ superfamily that signals via cell-surface tyrosine kinase receptors, is considered an essential neuroprotective ligand for midbrain dopaminergic neurons [1] with promising clinical trials in Parkinson's disease [2]. As GDNF has also been shown to promote the development and differentiation of dopaminergic neurons [3] perturbed regulation of its expression has been supposed to underlie several neuropsychiatric diseases such as schizophrenia and depression via dysregulation of dopaminergic neural circuitries and impaired synaptic plasticity [4], [5].

Analysis of GDNF level changes in depressive disorders revealed contradictory results. Both elevated [11], [12] and reduced [13], [14] GDNF plasma concentrations have been reported in patients with late-onset depression, major depression or bipolar disorder. Antidepressants and electroconvulsive therapy seemed to enhance rat hippocampal [15] and human plasma GDNF levels [16], [17] possibly via altered epigenetic regulation of the GDNF promoter [18]. On the other hand, a recent post mortem analysis of human brain samples disclosed elevated GDNF protein levels in the parietal cortex but not in limbic areas and basal ganglia of patients with depressive disorder [19].

Recent genetic association studies on neurotrophic factors investigated the brain-derived neurotrophic factor (BDNF) and the neurotrophin-3 receptor gene demonstrating association with depression [6], anxiety disorders [7], [8] or attention deficit hyperactivity disorder [9]. Evidence was also provided for an interaction between dopaminergic (COMT) and neurotrophic (BDNF) gene variants influencing dysfunctional beliefs such as threat [10] which might be linked to anxiety. Interestingly, the potential etiopathological involvement of GDNF has rarely been addressed by genetic studies. A genome-wide linkage study has first shed light on the GDNF as potential candidate gene in schizophrenia [20], followed by contradictory results from case-control association studies [21], [22]. This issue has extensively been investigated later by Williams and co-workers [23]. They analyzed 9 SNPs (single nucleotide polymorphisms) encompassing the entire genetic locus as well as a poly-AGG repeat in the 3′ untranslated region, but neither of them proved to be significantly associated with schizophrenia. No associations have been found between *GDNF* SNPs and attention deficit hyperactivity disorder (ADHD) either [24], [25].

*GDNF* was also shown to have a protective effect against methamphetamine induced dopamine depletion related neurotoxicity [26]. In addition, a single nucleotide polymorphism (SNP) of *GDNF* (rs2910704) has recently been associated with methamphetamine dependence in a Japanese population [27]. Since mood disorders are often accompanied by drug abuse [28], and impaired dopaminergic signaling is a well-known factor in the pathogenesis of depression, we raised the question whether *GDNF* gene variants might be risk factors of depression or anxiety. To clarify this issue, here we present an association analysis between eight SNPs of the *GDNF* gene and mood characteristics assessed by the Hospital Anxiety and Depression Scale (HADS) questionnaire using data from 708 healthy Caucasians. To our best knowledge, this is the first study addressing the role of *GDNF* polymorphisms in anxiety and depression.

## Subjects and Methods

### Sample

Non related Caucasian (Hungarian) subjects participated on a voluntary basis from several educational facilities. They were recruited at the Institute of Psychology, Eötvös Loránd University. The study protocol was designed in accordance with guidelines of the Declaration of Helsinki, and was approved by the Scientific and Research Ethics Committee of the Medical Research Council (ETT TUKEB). The participants signed a written informed consent, provided buccal samples and filled out the Hospital Anxiety and Depression Scale (HADS). Selection criteria included no past or present psychiatric history (based on self-report), age between 18–35 years, valid *GDNF* SNP data for at least five of the eight analyzed SNPs and valid self-report data for the HADS subscales. A total of 837 independent samples were genotyped by the Open Array system, of which 767 subjects were between 18–35 years and 760 of them filled out the HADS self-report scale. All of these 760 subjects provided answers for at least six out of seven items in each HADS subscale, therefore valid scale data could be calculated. 708 of them had 5 or more GDNF genotypes providing the final study population. As a result, we analyzed data from 708 subjects (46.3% males, 53.7% females; mean age: 21.3±3.4 years). The sample comprised of 169 university students from the Institute of Psychology, Eotvos Lorand University, 217 college and university students from two law enforcement institutions in the Budapest area and 322 volunteers recruited on different occasions popularizing our research. Anxiety and depression scores (see Table S1 in File S1), as well as age and sex ratios (see Table 1) were different in these subgroups. On the other hand, genotype frequencies of GDNF SNPs were similar in the subgroups (see Table S2 in File S1), therefore the total sample was used for association analyses.

**Table 1:** Anxiety and depression in the three subject groups.

Subject groups	N	Age	Male/female
Psychology students	169	18–35 (20.32±2.74)	16.6%/83.4%
Students in law enforcement	217	18–35 (20.27±2.08)	73.3%/26.3%
Other volunteers	322	18–35 (22.57±3.96)	43.5%/56.5%
Total sample	708	18–35 (21.33±3.39)	46.3%/53.7%

**Note.** Range, mean values and StDev are provided for age.

### Phenotype measures

All subjects completed the Hungarian version [29] of the Hospital Anxiety and Depression Scale (HADS). This self-report tool was originally developed by Zigmond and Snaith [30]. The questionnaire contains 14 intermixed items of two scales for detecting levels of anxiety (7 items) and depression (7 items). Both scales contain straightforward and reversed items to ensure attentive responses. Items are scored from 0–3 based on the releated response category (e.g. most of the time – not at all). The final raw score of both scales range from 0–21, sum of the appropiate items' scores. In the paper describing the Hungarian translation and validation of the HADS questionnaire [29] high internal consistency and discriminating power was found based on a sample of 715 Hungarian cancer patients. Concurrent validity of the HADS depression and anxiety scales has been attested with the Symptom List and the Beck Depression Scale. HADS anxiety scores increased with the number of anxiety-related emotional problems, such as ‘fears’, ‘nervousness’, and ‘worry’ and similarly increased HADS depression scores were found in those reporting ‘depression’ and ‘sadness’. Correlation of the depression scale of the HADS and the Beck Depression Scale (r = 0.81) also indicate sufficient concurrent validity.

### The SNP selection criteria

Single nucleotide polymorphisms (SNPs) with a minor allele frequency (MAF) greater than 0.05 were selected from the Single Nucleotide Polymorphism database of NCBI (dbSNP). The pairwise tagging method using r2 threshold of 0.8 by Haploview was used to determine tagging SNPs based on HapMap data to obtain a proper coverage of the *GDNF* gene. SNPs with a reference from previous association studies concerning neuropsychiatric disorders were preferred.

### Sample preparation and SNP genotyping

Collection of buccal swabs and isolation of genomic DNA was carried out as described in [24] with some modifications. Briefly, swabs were incubated in 450 µL lysis solution containing 0.2 g/L Proteinase K, 0.1 M NaCl, 0.5% SDS and 0.01 M Tris buffer, pH  = 8 at 56°C overnight followed by RNase treatment at room temperature. Proteins were removed with saturated NaCl (2∶1 volume ratio). After the standard procedure of DNA precipitation with isopropanol and ethanol, the pellet was resuspended in 100 µL of 5 mM Tris pH  = 8, 0.5 mM EDTA. Concentration of double stranded DNA was measured by fluorometry applying an intercalation assay (AccuBlue Broad Range dsDNA Quantification Kit, Biotium, Hayward). The range of the DNA concentration was 15–200 ng/µL, samples with lower than 15 ng/µL were not used for the OpenArray analysis.

Genotypes were determined applying the TaqMan^®^ OpenArray™ Genotyping System (Applied Biosystems, Forster City, CA) based on sequence-specific, fluorescent TaqMan probes in combination with a high-throughput PCR system using nanoliter-scale sample volume and post-PCR (endpoint) detection. Genotyping platforms were obtained from the manufacturer as immobilized target specific primers and fluorescent probes in a low density array format. Reaction mixtures containing approximately 100 ng DNA (range: 30–150 ng) and the 1× master mix (each deoxyribonucleoside triphosphate and the AmpliTaq Gold DNA-polymerase, provided by the manufacturer) were prepared on a 384-well sample plate and then loaded on the genotyping plates by the OpenArray™ Autoloader. PCR amplification was performed in the GeneAmp^®^ PCR System 9700 (Applied Biosystems, Forster City, CA) following the manufacturer's instruction. Endpoint imaging of the allele specific FAM and VIC fluorescent intensities was made by the OpenArray™ NT Imager. Raw data were evaluated by the TaqMan Genotyper v1.2 software.

2% of the DNA samples were repeatedly applied on the OpenArray system, demonstrating a 98,2% reproducibility. In addition, a subsample was re-genotyped for two SNPs (rs3812047, rs3096140) were re-genotyped with a 7300 Real-Time PCR System (Applied Biosystems, Foster City, CA) for quality control also providing an increase in the call rate of these SNPs. Original call rates of OpenArray™ Genotyping System are presented in Table S3 in File S1, while the call rates calculated for the final study population is given in Table 2. As it can be seen in these Tables, all the genotypes were in Hardy-Weinberg equilibrium.

**Table 2:** Genotype distribution of the studied *GDNF* polymorphisms.

dbSNP No	Genotype	N	%	HWE*	Call rate
rs1981844	GG	313	55.1	p = 0.537	80.2%
	CG	224	39.4		
	CC	31	5.5		
rs3812047	GG	542	76.6	p = 0.962	100,0%
	GA	154	21.7		
	AA	12	1.7		
rs3096140	TT	316	48.0	p = 0.928	92.9%
	TC	283	43.0		
	CC	59	9.0		
rs2973041	AA	488	70.6	p = 0.727	97.6%
	AG	182	26.4		
	GG	21	3.0		
rs2910702	AA	380	54.8	p = 0.911	98.0%
	GA	270	38.9		
	GG	44	6.3		
rs1549250	TT	235	33.5	p = 0.970	99.0%
	TG	339	48.4		
	GG	127	18.1		
rs2973050	CC	237	40.6	p = 0.340	82.3%
	TC	282	48.4		
	TT	64	11.0		
rs11111	AA	535	75.9	p = 0.321	99,6%
	AG	153	21.7		
	GG	17	2.4		

**Note.** * Hardy-Weinberg equilibrium.

### Statistical Analysis

Statistical analyses were carried out using SPSS 20.0 for Windows. Chi-square analysis was used to test reliability of the measured genotype and allele frequencies. Lewontin's *D*' as well as *R* ^2^ values of linkage disequilibrium were determined using HaploView 4.2 [31]. Haplotypes were determined by the Phase program [32]–[34]. Independent-Samples t-test was used to assess sex differences; relationship with age has been tested by correlation analyses. One way analyses of covariance (ANCOVA) was used to test genetic associations of the single and multiple marker analyses in an allele-wise design. False positive results were ruled out by Bonferroni correction for multiple testing. The corrected level of significance was p<0.00313, as the nominal p (value 0.05) was divided by the number of analyses performed (8 SNPs ×2 HADS scales  = 16). Two-way ANOVA was used for testing the effect of prior associations in males and females.

Genotypic and phenotypic data of the present study is publicly available through the NCBI dbGaP data repository: http://www.ncbi.nlm.nih.gov/gap.

## Results

### Reliability of the tested phenotypes and genotypes

Chronbach Alpha values were calculated to test the internal consistency of the self-report phenotypes. In the present sample reliability coefficients were satisfactory for both Anxiety (0.75) and Depression (0.68) scales. The Pearson's correlation coefficient was used to assess inter-correlation of the two scales: r = 0.54 (p<0.0001). Mean score of the anxiety scale was 5.80 (±3.54), with individual scores ranging from 0 to 19. Mean depression score was 2.75 (±2.55), with a range from 0 to 16. All polymorphisms were in Hardy-Weinberg equilibrium [35] as the p-values presented in Table 2 showed no significant differences between the distribution of observed and calculated genotype frequencies.

### Age and sex as possible confounds

For testing sex differences on the two HADS scales Independent-Samples t-test was applied. Females showed significantly higher anxiety scores then males (6.47 compared to 5.02; t(706)  = –5.55; p<0.001), thus sex was used as a covariant in all association analyses. Depression scores showed no significant sex difference. There was no significant correlation between HADS scales and age. This might be due to the relatively narrow age-range in our sample, as 90% of participants were between 18–25 years of age.

### Association analyses of mood characteristics and *GDNF* polymorphisms

Table 3 summarizes results from the single marker analysis in both mood dimensions using one-way ANCOVAs with one of the *GDNF* SNPs as the grouping variable, with the HADS anxiety or the depression scale as the dependent variable and with sex as covariant. Association results for the 8 *GDNF* SNPs are represented in each row with the number of detected alleles, calculated MAF values, mean and standard deviation of anxiety and depression scores for carriers of the presented alleles and the corresponding p values from the ANCOVAs. Four SNPs (rs3812047, rs3096140, rs2910702, rs1549250) were associated with anxiety; scores were higher in the presence of the minor allele in all four cases. Corresponding statistical values for the above four SNPs labeled in bold in Table 3 were [F(1,1413) = 11.541, p = 0.0007, η2 = 0.008, power = 0.924]; [F(1,1313) = 10.282, p = 0.00138, η2 = 0.008, power = 0.893]; [F(1,1385) = 8.527, p = 0.00356, η2 = 0.006, power = 0.831]; and [F(1,1399) = 6.252, p = 0.01252, η2 = 0.004, power = 0.705], respectively. One *GDNF* SNP (rs1981844) showed association with the HADS depression scale, with higher scores in the presence of the minor allele [F(1,1133) = 5.086, p = 0.02431, η2 = 0.004, power = 0.615]. After correction for multiple testing, association of anxiety with rs3812047 and rs3096140 remained significant, labeled by single stars in Table 3. Mean anxiety was significantly higher in the presence of the minor (A) allele of the rs3812047 (6.68±3.72) as compared to the mean anxiety of major (G) allele carriers (5.68±3.50). The minor (C) allele of the rs3096140 was also a genetic risk factor of anxiety, as mean scores in the presence of this allele were higher (6.29±3.48) as compared to those with the major (T) allele (5.59±3.55). Both *GDNF* SNPs explained 0.8% of the variability of anxiety. Although the risk allele for anxiety was the minor allele for both SNPs, the number of participants in the study provided enough data points for association analyses with sufficient power (for rs3812047 MAF = 12.6%, N = 178; for rs3096140 MAF = 30.5%, N = 401).

**Table 3:** Association of *GDNF* polymorphisms and mood dimensions.

Association analysis							
dbSNP No	alleles	N	MAF**	Anxiety	p	Depression	p
rs1981844	C	286	0.252	6.16 (±3.87)	0.07491	3.04 (±2.83)	0.02431
	G	850		5.71 (±3.41)		2.64 (±2.53)	
**rs3812047**	A	178	0.126	6.68 (±3.72)	0.00070*	2.93 (±2.55)	0.32941
	G	1238		5.68 (±3.50)		2.73 (±2.55)	
**rs3096140**	C	401	0.305	6.29 (±3.48)	0.00138*	2.80 (±2.51)	0.76512
	T	915		5.59 (±3.55)		2.75 (±2.54)	
rs2973041	G	224	0.162	5.64 (±3.81)	0.52321	3.01 (±2.81)	0.08250
	A	1158		5.80 (±3.50)		2.69 (±2.51)	
rs2910702	G	358	0.258	6.27 (±3.62)	**0.00356**	2.83 (±2.58)	0.59415
	A	1030		5.60 (±3.52)		2.74 (±2.56)	
rs1549250	G	593	0.423	6.07 (±3.68)	**0.01252**	2.90 (±2.67)	0.05343
	T	809		5.56 (±3.38)		2.63 (±2.43)	
rs2973050	T	410	0.352	6.11 (±3.63)	0.05656	2.85 (±2.69)	0.39771
	C	756		5.65 (±3.50)		2.72 (±2.54)	
rs11111	G	187	0.133	5.78 (±3.86)	0.84093	2.97 (±2.67)	0.22126
	A	1223		5.79 (±3.49)		2.72 (±2.54)	

**Notes.** *Significant after Bonferroni correction (p<0.00313) in single marker analyses. **MAF: minor allele frequency.

Since the law enforcement subgroup of our sample showed markedly lower anxiety mean scores than the subgroup of psychology students and other volunteers, we carried out two post-hoc analyses testing association of anxiety and the rs3812047 and rs3096140 GDNF polymorphisms without the law enforcement subgroup. Omitting this subgroup did not alter our previous findings using the total sample, the same pattern of risk alleles for increased anxiety was demonstrated: Mean anxiety score was higher (7.43±3.56) in the presence of the rs3812047 A allele as compared to 6.22(±3.38) in those carrying the rs3812047 G allele [F(1,903) = 12.358, p = 0.00046, η2 = 0.014, power = 0.940]. Similarly, in the presence of the rs3096140 C allele mean anxiety level was higher (6.74±3.25) than in the presence of the rs3096140 T allele (6.17±3.50), [F(1,837) = 4.682, p = 0.03077, η2 = 0.006, power = 0.580]. According to these results association of rs3812047 and rs3096140 GDNF polymorphisms with anxiety was consistent across participant subgroups.

### Haplotype analysis

**Figure 1:**
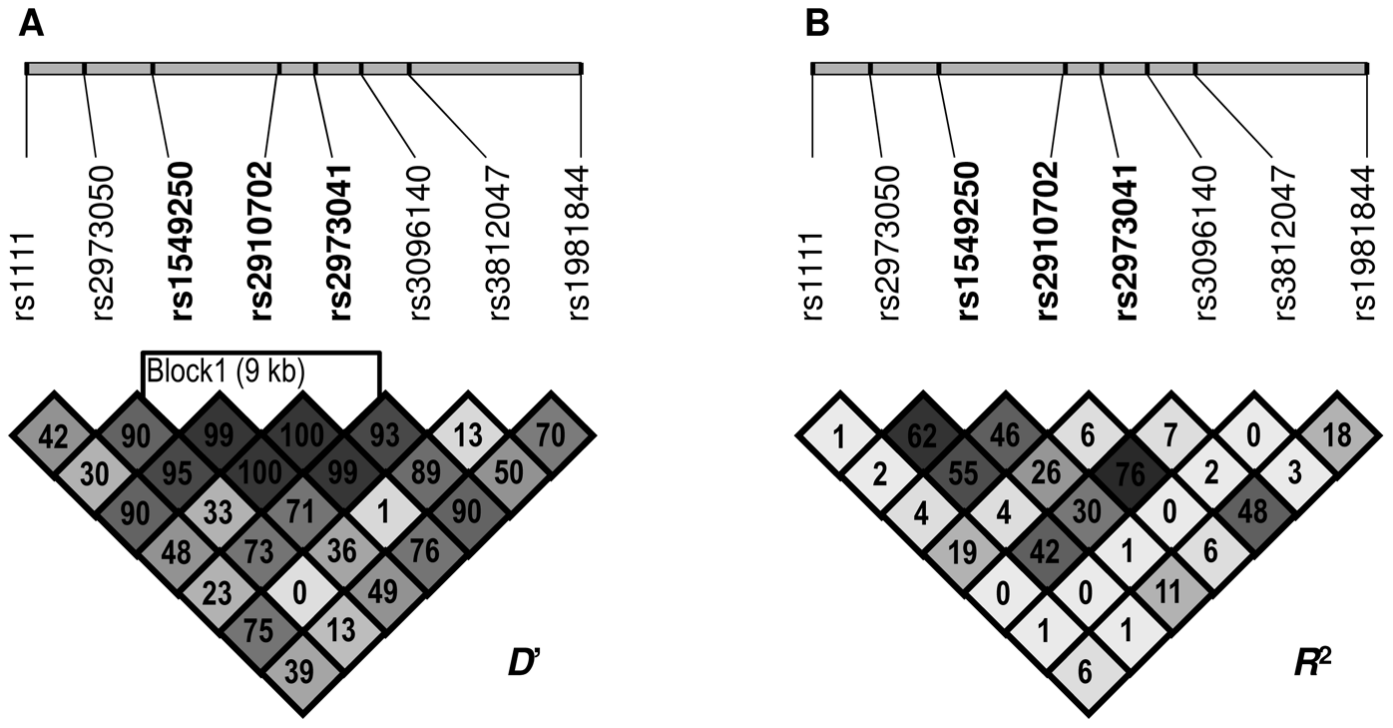
Linkage disequilibrium plots for the studied *GDNF* SNPs. A: Lewontin's D' measure and B: R2 values of linkage disequilibrium. Higher values and darker squares indicate stronger pairwise linkage disequlibrium between two loci.

As the two SNPs (rs3812047 and rs3096140) significantly associated with anxiety after correction for multiple testing were not in linkage disequilibrium (D' = 13, r^2^ = 0; see Figure 1A and 1B), haplotype analysis was also performed. One-way ANCOVAs were applied on the two mood dimensions with the haploalleles as the grouping variable and sex as covariant (results are presented in Table 4). Effect of haplotypes on anxiety was significant [F(1,1415) = 6.323, p = 0.00029, η^2^ = 0.013, power = 0.967], while there was no significant differences in the mean scores of depression. Mean anxiety was lowest when formerly defined risk alleles were not present in the in the haplotype (5.49±3.45). When rs3096140C was present in the haploallele mean anxiety scores were notably higher (6.08±3.57). Anxiety scores were even higher when rs3812047A was present in the haploallele (6.50±4.04), and anxiety reached its highest average in the presence of both risk alleles in the haplotype (7.09±2.80).

**Table 4:** Haplotype analysis of risk alleles.

Haplotypes*	N	MAF**	Anxiety	p	Depression	p
rs3812047G_rs3096140T	853	0,602	5.49 (±3.45)	**0.00029**	2.72 (±2.60)	0.804
rs3812047G_rs3096140**C**	385	0,272	6.08 (±3.57)		2.74 (±2.46)	
rs3812047**A**_rs3096140T	125	0,088	6.50 (±4.04)		2.90 (±2.53)	
rs3812047**A**_rs3096140**C**	53	0,037	7.09 (±2.80)		2.98 (±2.64)	

Notes. *Risk alleles in the haplotypes are labeled by bold. **MAF: minor allele frequency.

### Effect of *GDNF* risk alleles on male and female anxiety

**Figure 2:**
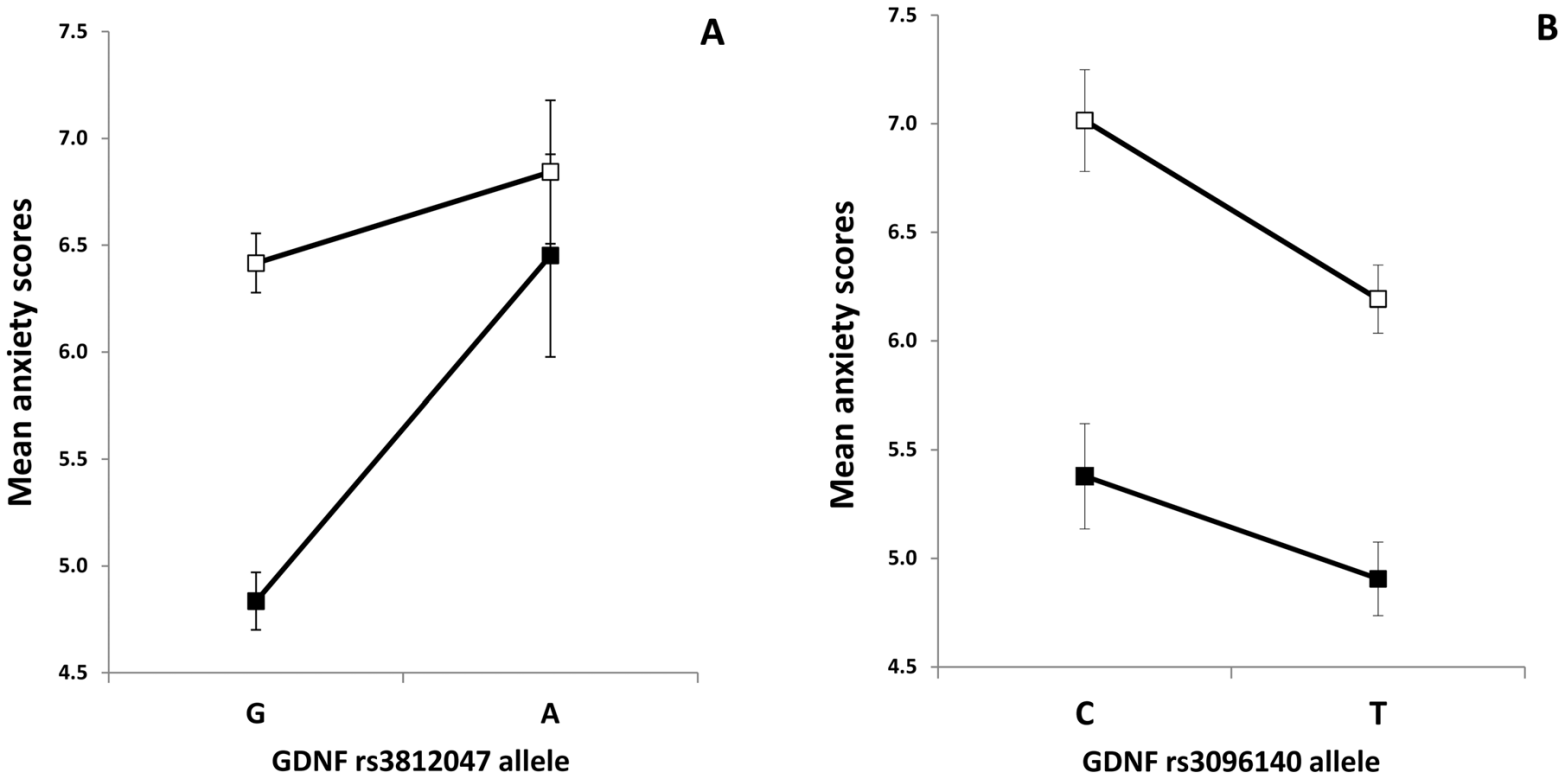
Effect of *GDNF* risk alleles on male and female anxiety. Mean HADS anxiety scores in females and males as a function of rs3812047 (A) and rs3096140 (B) alleles. Open markers denote females; closed markers denote males. Error bars represent standard errors of the mean.

There was a significant sex difference in HADS anxiety scores. In order to test if the significant genetic effects from our single marker analyses were different for males and females we used two-way ANOVAs on anxiety as the dependent variable and sex and presence or absence of the risk allele of one of the two SNP as grouping factors. Results are presented in Figure 2A and 2B. Main effect of the rs3812047 SNP was significant [F(1,1412) = 13.391, p = 0.0002, η2 = 0.009, power = 0.955] and as expected, we found a significant main effect of sex [F(1,1412) = 12.48, p = 0.0004, η2 = 0.009, power = 0.942]. Interestingly a significant interaction between sex and the rs3812047 SNP was also observed [F(1,1412) = 4.539, p = 0.033, η2 = 0.003, power = 0.567]. The effect of the minor (A) allele as a risk for higher anxiety was more pronounced in males as compared to females (Figure 2A).

Main effect of the rs3096140 SNP was also significant on anxiety [F(1,1312) = 9.664, p = 0.002, η2 = 0.007, power = 0.874], and we also found a significant sex effect [F(1,1312) = 49.233, p<0.0001, η2 = 0.036, power = 1.000]. However, for this *GDNF* SNP there was no significant gene-sex interaction. As presented in Figure 2B both males and females with the minor (C) allele showed higher anxiety.

## Discussion

Evidence from twin studies confirms that mood characteristics have a considerable genetic component. Heritability of major depression is 37% [36], [37] and heritability of affective and anxiety disorders is around 45% [38]. The association between mood disorders and the monoamine system, especially the dopamine pathways, is well established [39]–[41], however, there are no prior reports on the effect of *GDNF* polymorphisms on mood characteristics in clinical or non-clinical populations.

The aim of the present study was to investigate any possible association of the *GDNF* gene polymorphisms with non-clinical individual variations of anxiety and depression. Several methods have been proposed to date for measuring mood characteristics (for recent reviews see: [42], [43]. In the present study we used the HADS questionnaire which has been translated to several languages [44] and is applicable to measure anxiety and depression in somatic, psychiatric and non-clinical samples [45]. We also demonstrated applicability of the HADS questionnaire in our previous genetic association findings, e.g. reporting association between polymorphisms of the P2RX7 gene and depression scores of diabetic patients [46] and patients with major depression or bipolar disorder [47].

Here we explored the association of 8 *GDNF* polymorphisms with anxiety and depression (Table 3). After correcting for multiple testing, the genetic effect on depression did not remain significant; however, two of the studied *GDNF* SNPs (rs3812047A and rs3096140C) were identified as possible risk alleles of anxiety (level of significance for the two associations were p = 0.00070 and 0.00138, respectively). We replicated these association findings using a subsample without the law enforcement subgroup, since they showed markedly lower anxiety mean scores than the subgroup of psychology students and other volunteers. Mean anxiety scores were higher in the presence of the rs3812047 A and the rs3096140 C risk alleles. According to these results association of rs3812047 and rs3096140 GDNF polymorphisms with anxiety was consistent across participant subgroups.

Since the two risk-SNPs were not in linkage disequilibrium we also performed haplotype analysis of these SNPs. Results described in Table 4 underlie the significant genetic effect indicated by our single-marker analyses: mean anxiety scores raised according to the number of risk alleles present in the haplotypes (p = 0.00029). It should be noted that anxiety and depression scales of the HADS questionnaire correlate (r = 0.54, p<0.0001) implicating that these two constructs are in close relation. One possible reason for the lack of significant effects of *GDNF* polymorphisms on depression in the present study is that depression scores were quite low in our non-clinical sample. This floor effect [48] might have reduced individual variation, and diminished genetic effects.

Findings from previous studies also confirm sex differences in anxiety. Higher anxiety of females was consistent according to a meta-analysis [49] with studies using a wide range of subject pool and anxiety measures (e.g. State-Trait Anxiety Inventory, Children's Manifest Anxiety Scale, Minnesota Multiphasic Personality Inventory). Anxiety disorders are also more frequent in females and more anxiety symptoms characterize them [50]. Neurotransmitter systems probably play an important role in the background of these differences [51], for example through the estrogen system increasing monoamine synthesis (dopamine, serotonin, norepinephrine) and receptor sensitivity [52]. Our results support these findings, as female subjects reported higher anxiety scores. However, we also report an interaction effect of sex and rs3812047 SNP on anxiety (Figure 2A). Males with the minor (A) allele showed anxiety scores as high as females with the major (G) allele of this polymorphism. Females with the risk (A) allele reported even higher anxiety scores. Others [53] also reported interaction effect of sex and the BDNF Val66Met polymorphism on stress reactivity.

**Figure 3:**
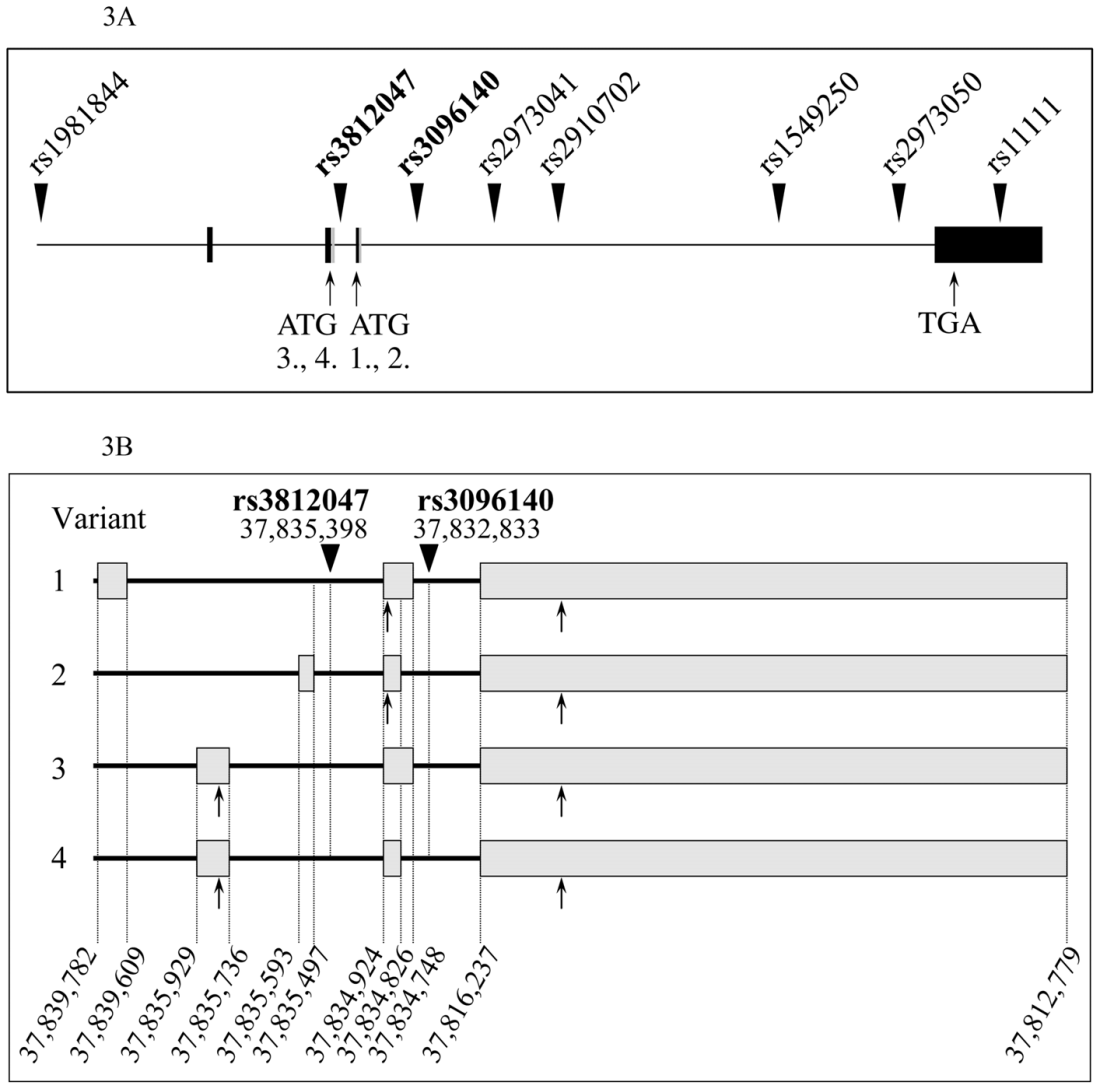
**A. Localization of the analyzed human *GDNF* gene polymorphisms.** Exons are labeled by filled boxes, the approximate position of the studied SNPs by filled triangles. SNPs showing significant association with anxiety after correction for multiple testing are in bold. ATG 1,2,3 and 4: alternative start sites, TGA: stop signal. **B. Fine map of the genomic location of GDNF isoforms.** Chromosomal positions of exons in each transcription variant are indicated at the bottom. (Please note, that introns are longer than they appear in the figure.) Arrows pointing up indicate the start and stop codons of open reading frames in each isoform. Chromosomal localization of rs3812047 and rs3096140 are shown on the top. Distance between exon 1 of variant 2 and rs3812047 is 99 bp, whereas exon 2 is 474 bp away from the polymorphic locus. Similarly, distance between exon 2 of isoform 1 or 3 and rs3096140 is 1915 bp, and exon 3 is 16596 bp away from the SNP.

Limitations of the presented study involve the relatively low sample size, therefore the possibility of false positive findings could not be excluded despite the fact that the two reported significant findings survived the Bonferroni-correction for multiple testing. Therefore further replications with independent samples are necessary. Moreover, no functional data are available concerning the intronic SNPs shown here to associate with anxiety, however, it is important to note that both anxiety-linked SNPs are in the close proximity of critical sites of alternative splicing (Figure 3.A. and B). Nevertheless, the fact that only these two SNPs associate firmly with anxiety might imply their significance in the control of gene expression. As far as the molecular background of this novel association is concerned, it is tempting to assume that these polymorphisms might be involved in the regulation of alternative splicing of the GDNF gene. Importantly, there are two main types of alternatively spliced preproGDNF isoforms, alpha and beta, the latter possessing a significantly shorter propeptide sequence due to the presence of an alternative splicing site in exon 3 of the gene [54], resulting in four isoforms as shown on Figure 3 B. Although the functional differences of GDNF variants are not fully understood, altered processing and secretion of the protein isoforms have been demonstrated [55]. This assumption seems highly probable in the light of recent publications assigning a pivotal role to intronic polymorphisms in governing splicing processes via differential recruitment of key splicing factors. For instance, two intronic SNPs in the type 2 dopamine receptor gene (DRD2) have been found sufficient to affect alternative splicing and therefore susceptibility to cocaine abuse [56]. Similar interactions between intronic SNPs and alternatively spliced isoforms have also been described in case of the human myocilin [57] and insulin [58] as well as the human papilloma virus E6/E7 genes [59], just to mention but a few.

Albeit several lines of biochemical evidence argue for the role of *GDNF* in dopaminergic differentiation [1], relatively scarce and ambiguous data have been gained from association studies with regard to its involvement in the pathogenesis of neuropsychiatric disorders. To date, a cohort of association analyses has suggested that certain *GDNF* polymorphisms might be linked to schizophrenia, a pervasive neurodevelopmental disorder [20], [23]. Recent findings from Ahmadiantehrani and Ron [60] seem to corroborate these results by revealing that upregulated DRD2 signaling, a hallmark of schizophrenia, resulted in elevated *GDNF* expression levels.

To our best knowledge, this is the first report shedding light on the significance of the rs3812047 and rs3096140 SNPs that have not been found to significantly associate with any known traits or disorders before. Previously, a study conducted on Japanese drug abusers identified a *GDNF* SNP associated with metamphetamine dependence [27]. It is widely known that anxiety disorders such as generalized anxiety disorder, phobias, panic- and compulsivity disorders are often accompanied by drug addiction, smoking and heavy drinking [61], [62]. In light of results presented here, *GDNF* might be one of the common factors that links anxiety to substance abuse.

This is the first report on association between anxiety and the polymorphisms of *GDNF* gene; however, since we used a non-clinical sample we could assess genetic background of individual variation in anxiety below the clinical threshold. Further studies are needed to reveal whether the genetic risk factors suggested here are related to higher vulnerability of mood disorders.

## Supporting Information

File S1 **Table S1.** Anxiety and depression in the three subject groups. **Table S2.** Genotype frequencies of GDNF SNPs in the three subject groups. **Table S3.** Technical data of genotypes obtained by the OpenArray™ Genotyping System.
(DOCX)
