# Supplementary material for: Glial Cell Line-Derived Neurotrophic Factor (GDNF) as a Novel Candidate Gene of Anxiety
Source: PLoS One. 2013 Dec 6;8(12):e80613. doi: 10.1371/journal.pone.0080613 (PMC3855631; doi:10.1371/journal.pone.0080613)
Supplement: File S1 — Table S1. Anxiety and depression in the three subject groups. Table S2. Genotype frequencies of GDNF SNPs in the three subject groups. Table S3. Technical data of genotypes obtained by the OpenArray™ Genotyping System. (DOCX) [file pone.0080613.s001.docx]

**Supplementary TABLE S1.**

**Anxiety and depression in the three subject groups**

| **Subject groups** | **N** | **Anxiety** | **Depression** |
| --- | --- | --- | --- |
| Psychology students | 169 | 0-18 (6.7±3.39) | 0-12 (2.9±2.39) |
| Students in law enforcement | 217 | 0-17 (4.2±3.19) | 0-16 (2.2±2.54) |
| Other volunteers | 322 | 0-19 (6.4±3.52) | 0-13 (3.0±2.60) |
| Total sample | 708 | 0-19 (5.8±3.54) | 0-16 (2.8±2.55) |

**Note.** Range, mean values and StDev are provided for HADS anxiety and depression scores.

**Supplementary TABLE S2.**

**Genotype frequencies of GDNF SNPs in the three subject groups**

| **dbSNP No.** | **Genotype** | **N (%) in the subject groups** | | | | | |
| --- | --- | --- | --- | --- | --- | --- | --- |
|  |  | Psychology students | | Students in law enforcement | | Other volunteers | |
| rs1981844 | GG | 73 | 57.9% | 91 | 51.7% | 149 | 56.0% |
|  | CG | 45 | 35.7% | 77 | 43.8% | 102 | 38.4% |
|  | CC | 8 | 6.4% | 8 | 4.5% | 15 | 5.6% |
| rs3812047 | GG | 122 | 72.2% | 168 | 77.4% | 252 | 78.3% |
|  | GA | 41 | 24.3% | 48 | 22.1% | 65 | 20.2% |
|  | AA | 6 | 3.5% | 1 | 0.5% | 5 | 1.5% |
| rs3096140 | TT | 71 | 43.8% | 106 | 52.5% | 139 | 47.1% |
|  | TC | 72 | 44.5% | 81 | 40.1% | 131 | 44.4% |
|  | CC | 19 | 11.7% | 15 | 7.4% | 25 | 8.5% |
| rs2973041 | AA | 123 | 75.5% | 149 | 69.0% | 216 | 69.2% |
|  | AG | 36 | 22.1% | 60 | 27.8% | 86 | 27.6% |
|  | GG | 4 | 2.4% | 7 | 3.2% | 10 | 3.2% |
| rs2910702 | AA | 88 | 52.7% | 124 | 58.5% | 168 | 53.3% |
|  | GA | 65 | 38.9% | 75 | 35.4% | 130 | 41.3% |
|  | GG | 14 | 8.4% | 13 | 6.1% | 17 | 5.4% |
| rs1549250 | TT | 60 | 35.9% | 76 | 35.2% | 99 | 31.1% |
|  | TG | 74 | 44.3% | 102 | 47.2% | 163 | 51.3% |
|  | GG | 33 | 19.8% | 38 | 17.6% | 56 | 17.6% |
| rs2973050 | CC | 52 | 40.6% | 82 | 46.1% | 103 | 37.2% |
|  | TC | 55 | 43.0% | 82 | 46.1% | 145 | 52.3% |
|  | TT | 21 | 16.4% | 14 | 7.8% | 29 | 10.5% |
| rs11111 | AA | 129 | 76.8% | 160 | 73.7% | 246 | 76.9% |
|  | AG | 34 | 20.2% | 54 | 24.9% | 65 | 20.3% |
|  | GG | 5 | 3.0% | 3 | 1.4% | 9 | 2.8% |

**Supplementary TABLE S3.**

**Technical data of genotypes obtained by the OpenArray™ Genotyping System**

| **dbSNP No.** | HWE***** | Call rate |
| --- | --- | --- |
| rs1981844 | p = 0.720 | 78.7% |
| rs3812047 | p = 0.860 | 93.1% |
| rs3096140 | p = 0.986 | 90.1% |
| rs2973041 | p = 0.746 | 94.6% |
| rs2910702 | p = 0.961 | 96.4% |
| rs1549250 | p = 0.999 | 97.3% |
| rs2973050 | p = 0.539 | 79.9% |
| rs11111 | p = 0.186 | 98.4% |

**Note. *** Hardy-Weinberg equilibrium
